# BUN level is associated with cancer prevalence

**DOI:** 10.1186/s40001-023-01186-4

**Published:** 2023-07-01

**Authors:** Cai Wang, Hao Sun, Jin Liu

**Affiliations:** 1 Binhai County People’s Hospital, Yancheng, 224500 Jiangsu China; 2 Shandong Tumor Hospital and Institute, Shandong First Medical University and Shandong Academy of Medical Sciences, Jinan, 250117 China

**Keywords:** BUN, Cancer prevalence, NHANES, Morbidity rate, Breast

## Abstract

Blood urea nitrogen (BUN) was an important biomarker for the development and prognosis of many diseases. Numerous studies had demonstrated that BUN had a strong relationship with long-term mortality, survival and the prevalence of some diseases. The diagnosis and treatment, prognosis and long-term survival rate of cancer were the focus of clinical research at present. However, the relationship between BUN level and cancer prevalence was not clear. To investigate the relationship between BUN level and cancer prevalence, we performed a statistical analysis of population data from the National Health and Nutrition Examination Survey (NHANES) database. The results of the study showed that BUN level were positively correlated with cancer prevalence, and the correlation was more pronounced in breast cancer.

## Introduction

Blood urea nitrogen (BUN) was a nitrogenous compound in plasma except protein, which was excreted by the kidney. The level of BUN was determined by the balance among urea production, metabolism and excretion [1]. The level of BUN was closely related to the occurrence and development of many diseases, long-term mortality and survival rate [2, 3]. BUN was an important indicator of renal metabolism and played an important role in diagnosis, treatment and prognosis in the occurrence and development of renal disease [4–7]. In a study of BUN level and the risk of insulin use, it was found that high BUN level increased the risk of insulin use [8]. Persistently high BUN level was associated with increased risk of cardiovascular death and readmission of heart failure [9]. In another study, it was found that the ratio of BUN/creatinine played an important role in predicting the prognosis of heart failure [10]. BUN/albumin ratio was an effective predictor of mortality in geriatric emergency department. The higher the ratio, the higher the risk of hospital mortality [11]. The increase of BUN level had predictive significance in the prognosis of acute ischemic stroke [12]. Studies by BoHu et al. had shown that the higher the level of BUN, the longer the hospital stay and the higher the mortality of patients with primary pulmonary hypertension [13].

Cancer is a complex malignant disease, and despite the continuous development of medical technology, the prevalence and mortality of cancer continue to rise [14]. Cancer was usually caused by a variety of internal and external factors, including genetic mutations, chemical factors, physical factors, psychological factors, etc. [15–17]. Therefore, more attention had been paid to the prediction of high risk factors inducing cancer, which played an important role in the early diagnosis and treatment of cancer [18–20].

In this work, we selected some people from the National Health and Nutrition Survey (NHANES) survey participants to study the relationship between BUN and cancer prevalence. We performed univariate and multivariate logistic regression analysis between BUN level and cancer prevalence. In addition, we also analyzed the relationship between BUN level and the occurrence of different types of cancer, as well as subgroup analysis. The results showed that people with high level of BUN had a higher risk of cancer, especially breast cancer.

## Materials and methods

### Study population

**Fig. 1:**
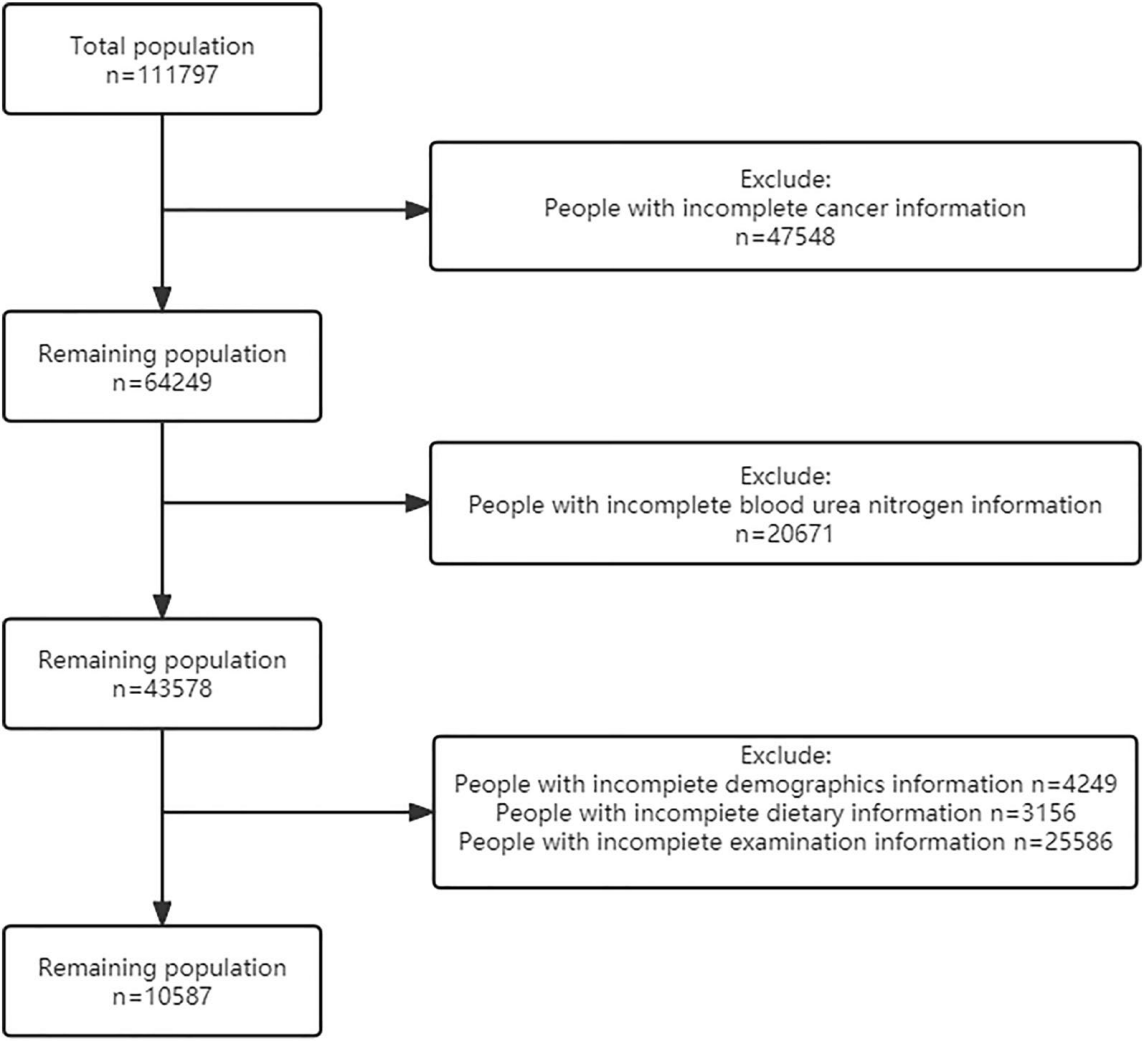
Screening conditions and process for the study population

Data on the study population were obtained from the National Health and Nutrition Inspection Survey (NHANES) database [21, 22]. Our study included 10,587 NHANES participants, representing 71,672,324 Americans, with an overall weighted prevalence of cancer of 10.65%. The survey was approved by the Institutional Review Board of the National Center for Health Statistics, and all patients gave informed consent. The selection flowchart of the study population is shown in Fig. 1. First of all, the population with incomplete cancer information was excluded from the total population (*n* = 111,797), and the remaining population was 64,249. After that, people with incomplete BUN level information were excluded from the rest of the population. In the end, among the remaining 43,578 people, we excluded people with incomplete demographic information (*n* = 4249), incomplete dietary information (*n* = 3156), and incomplete examination information (*n* = 25,586).

### Variables

#### Demographic characteristics

Data were obtained on age (> 65/< 65), sex (male/female), race (non-Hispanic White, non-Hispanic Black, Mexican American, Other Hispanic, other race), education level (less than high school, high school, more than high school).

#### Smoking

We divided the population according to the frequency of smoking. The smoking behavior of participants was divided into: never (smoked less than 100 cigarettes in life), former (smoked more than 100 cigarettes in life and smoke not at all now), now (smoked more than 100 cigarettes in life and smoke some days or every day).

#### Drinking

The drinking habits of the people who participated in the survey were divided into: never (had < 12 drinks in lifetime), former (had ≥ 12 drinks in 1 year and did not drink last year, or did not drink last year but drank ≥ 12 drinks in lifetime), mild (≤ 1 drink per month for women and ≤ 2 drinks per month for men), moderate (≤ 2 drinks per month for women and ≤ 3 drinks per month for men), and heavy (≥ 3 drinks per month for women and ≥ 4 drinks per month for men).

#### Metabolic disease

Diagnostic criteria of hypertension: blood pressure ≥ 140/90 mmHg. Average blood pressure was calculated by the following protocol: (1) the diastolic reading with zero was not used to calculate the diastolic average; (2) if all diastolic reading were zero, then the average would be zero; (3) if only one blood pressure reading was obtained, that reading was the average; (4) if there was more than one blood pressure reading, the first reading was always exclude from the average.

The diagnostic criteria for diabetes were as follows: (1) doctor told you have diabetes; (2) glycohemoglobin HbA1c (%) > 6.5; (3) fasting glucose (mmol/l) ≥ 7.0; (4) random blood glucose (mmol/l) ≥ 11.1; (5) 2-h OGTT blood glucose (mmol/l) ≥ 11.1; (6) use of diabetes medication or insulin.

#### Moderate exercise time

Definition of moderate exercise: moderate exercise, fitness, or recreational activity can lead to a slight increase in breathing or heart rate for at least 10 min.

#### Nutritional condition

The nutritional status data of the patients were analyzed according to the results of property income ratio (PIR), body mass index (BMI), waist circumference and energy intake. The cut-off value of PIR was 2.9%. The results of BMI were classified as > 25 kg/m^2^ or ≤ 25 kg/m^2^. The value of waist circumference was divided by 97 cm. The energy intake value was the average of the total energy intake in 2 days, and the daily energy intake came from the total energy of food and beverages. The mean value of total energy intake for both days was classified as > 1950 kcal or ≤ 1950 kcal.

#### Cancer site

According to the location and tissue location of cancer, the types of cancer were divided into: breast cancer, melanoma, prostate cancer, uterine cancer, skin cancer, colon cancer and others. For the accuracy of the experiment, we ruled out small cancers with a lower incidence.

### Statistical analysis

The filtered data were analyzed using R (version 4.2.1). Before starting the analysis, we weighted the data. For continuous variables, we used *x*^−^ (95% CI) for statistical description and *t*-test for comparison between groups. For categorical variables, we used *p* (95% CI) for statistical description and Chi-square test for comparison between groups. For adjusted analysis, we chose binary logistic regression for multifactorial analysis. To investigate the relationship between BUN and different types of cancer, we selected several cancers with high prevalence (breast, colon, melanoma, prostate, cervical, and skin) for logistic regression analysis. In addition, we adjusted the data for subgroups by binary logistic regression for the analysis. Bilateral *P* < 0.05 was considered a statistically significant difference.

## Results

### Characteristics of the study population

The baseline characteristics of the population studied in the experiment are shown in Table 1. The prevalence of cancer was statistically different among age, race, PIR, smoking, drinking level, waistline, energy intake, hypertension and diabetes.

**Table 1:** Baseline characteristic table of the study population

Characteristics	Cancer		*P* value
	No	Yes	
Total	89.35 (84.35, 94.34)	10.65 (9.77, 11.54)	
Age ~ %			< 0.01
< 65	86.96 (85.91, 88.01)	55.27 (50.88, 59.67)	
≥ 65	13.04 (11.99, 14.09)	44.73 (40.33, 49.12)	
Gender ~ %			0.59
Male	48.31 (47.05, 49.57)	46.27 (42.09, 50.44)	
Female	51.69 (50.43, 52.95)	53.73 (49.56, 57.91)	
Race ~ %			< 0.01
Non-Hispanic White	72.46 (70.11, 74.82)	89.28 (87.20, 91.35)	
Non-Hispanic Black	8.76 (7.66, 9.85)	3.81 (2.95, 4.66)	
Mexican American	6.58 (5.46, 7.70)	1.38 (0.89, 1.87)	
Other Hispanic	4.79 (4.05, 5.52)	2.10 (1.09, 3.12)	
Other race	7.42 (6.59, 8.24)	3.44 (2.02, 4.85)	
Education level ~ %			0.06
Less than high school	7.19 (6.39, 7.99)	5.12 (3.40, 6.84)	
High school	18.99 (17.67, 20.31)	17.88 (14.81, 20.95)	
More than high school	73.82 (72.07, 75.57)	77.00 (73.26, 80.74)	
Family PIR ~ %	3.41 (3.33, 3.49)	3.78 (3.66, 3.91)	< 0.01
BMI ~ kg/m^2^	28.41 (28.20, 28.62)	28.34 (27.90, 28.77)	0.76
Smoking behavior ~ %			< 0.01
Never	60.94 (59.25, 62.62)	51.53 (48.13, 54.93)	
Former	24.75 (23.36, 26.14)	39.70 (35.90, 43.50)	
Now	14.31 (13.27, 15.36)	8.77 (6.70, 10.84)	
Alcohol consumption ~ %			< 0.01
Never	8.74 (7.56, 9.93)	5.58 (4.05, 7.11)	
Former	8.34 (7.48, 9.21)	9.42 (7.48, 11.37)	
Mild	40.85 (39.08, 42.63)	55.92 (51.76, 60.07)	
Moderate	20.82 (19.65, 21.98)	17.16 (14.00, 20.31)	
Heavy	21.24 (19.97, 22.51)	11.93 (9.35, 14.50)	
Moderate exercise ~ min	64.32 (62.69, 65.95)	63.18 (58.92, 67.43)	0.62
Waist ~ cm	97.36 (96.82, 97.90)	99.76 (98.56, 100.97)	< 0.01
Energy intake ~ kcal	2127.60 (2102.28, 2152.93)	2029.08 (1965.78, 2092.38)	< 0.01
Hypertension ~ %			< 0.01
Yes	30.11 (28.67, 31.55)	50.38 (46.26, 54.50)	
No	69.89 (68.45, 71.33)	49.62 (45.50, 53.74)	
Diabetes ~ %			< 0.01
Yes	9.92 (9.20, 10.64)	19.37 (16.42, 22.33)	
No	90.08 (89.36, 90.80)	80.63 (77.67, 83.58)	
Blood urea nitrogen ~ mg/dL	13.58 (13.42, 13.75)	15.64 (15.21, 16.07)	< 0.01

### The relationship between BUN level and cancer prevalence

In order to further study the relationship between BUN level and cancer, we conducted univariate and multivariate logistic regression analysis (Table 2). Crude, an unmodified model, showed a positive association between BUN level and cancer prevalence (OR: 1.08, 95% CI 1.07–1.10) with a statistically significant difference (*P* < 0.01) in a univariate logistic regression analysis. Model 1 was adjusted for age and race and showed a positive association between BUN level and cancer prevalence (OR: 1.03, 95% CI 1.01–1.05), with a statistically significant difference (*P* < 0.01). Model 2 was adjusted for age, race, PIR, smoking, alcohol consumption, waist circumference and energy intake, and showed a positive association between BUN level and cancer prevalence (OR: 1.03, 95% CI 1.01–1.04), with a statistically significant difference (*P* = 0.01). Model 3 was adjusted for age, race, PIR, smoking, alcohol consumption, waist circumference, energy intake, hypertension, and diabetes, and showed no statistically significant difference between BUN level and cancer prevalence (*P* = 0.05). Model 4 was adjusted for age, race, PIR, smoking, alcohol consumption, waist circumference, energy intake, hypertension, diabetes, gender, education, BMI, and duration of moderate exercise, and the results showed a positive association between BUN level and cancer prevalence (OR: 1.02, 95% CI 1.00–1.04), with a statistically significant difference (*P* = 0.04).

**Table 2:** Results of the regression analysis between BUN level and cancer prevalence analyzed by different models

Outcomes	Model	OR (95% CI)	*P* value
Cancer	Crude	1.08 (1.07, 1.10)	< 0.01
	Model 1	1.03 (1.01, 1.05)	< 0.01
	Model 2	1.03 (1.01, 1.04)	0.01
	Model 3	1.02 (1.00, 1.04)	0.05
	Model 4	1.02 (1.00, 1.04)	0.04

### The relationship between BUN level and cancer species

**Fig. 2:**
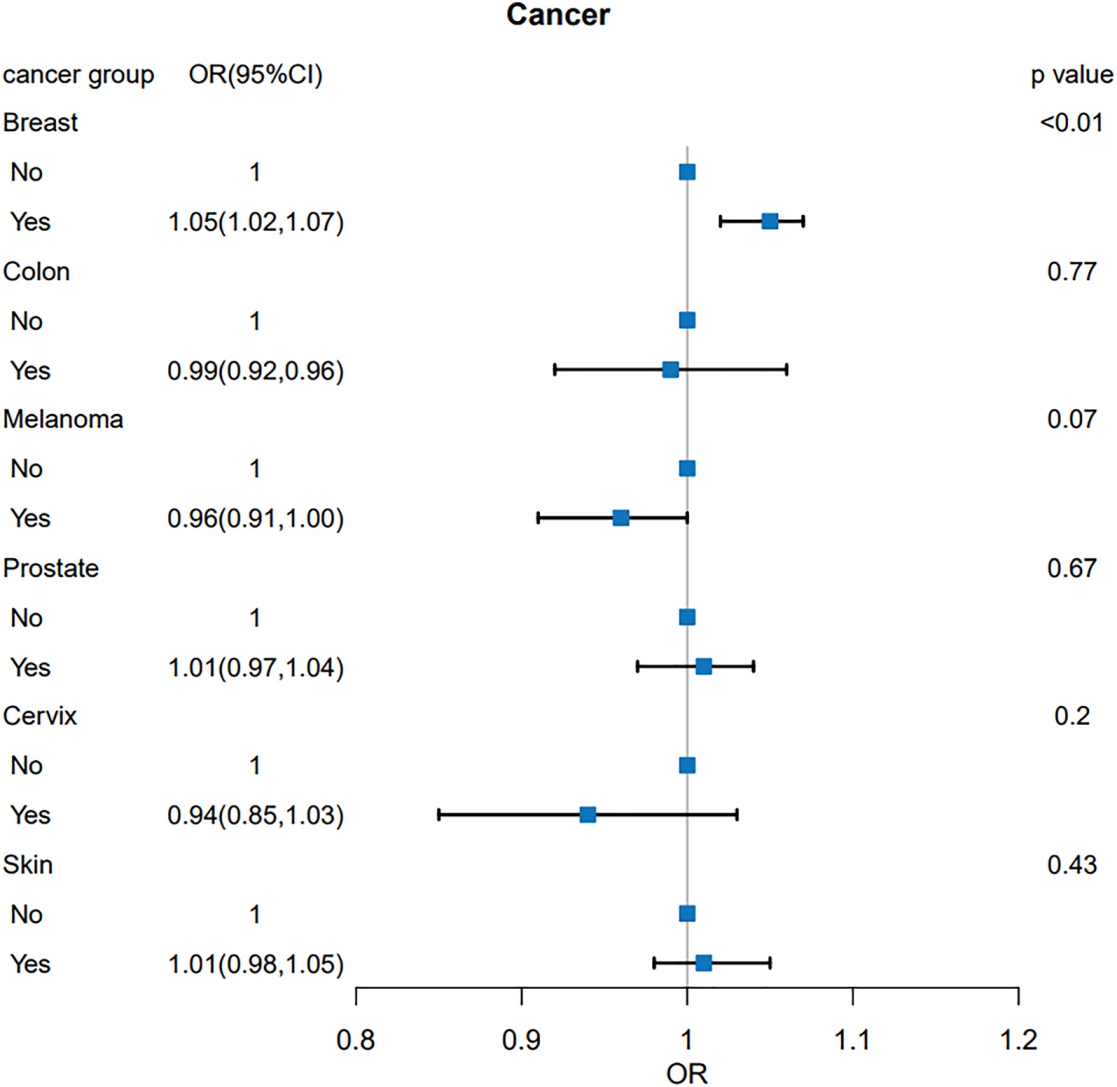
Results of regression analysis of the relationship between BUN level and different cancers

**Fig. 3:**
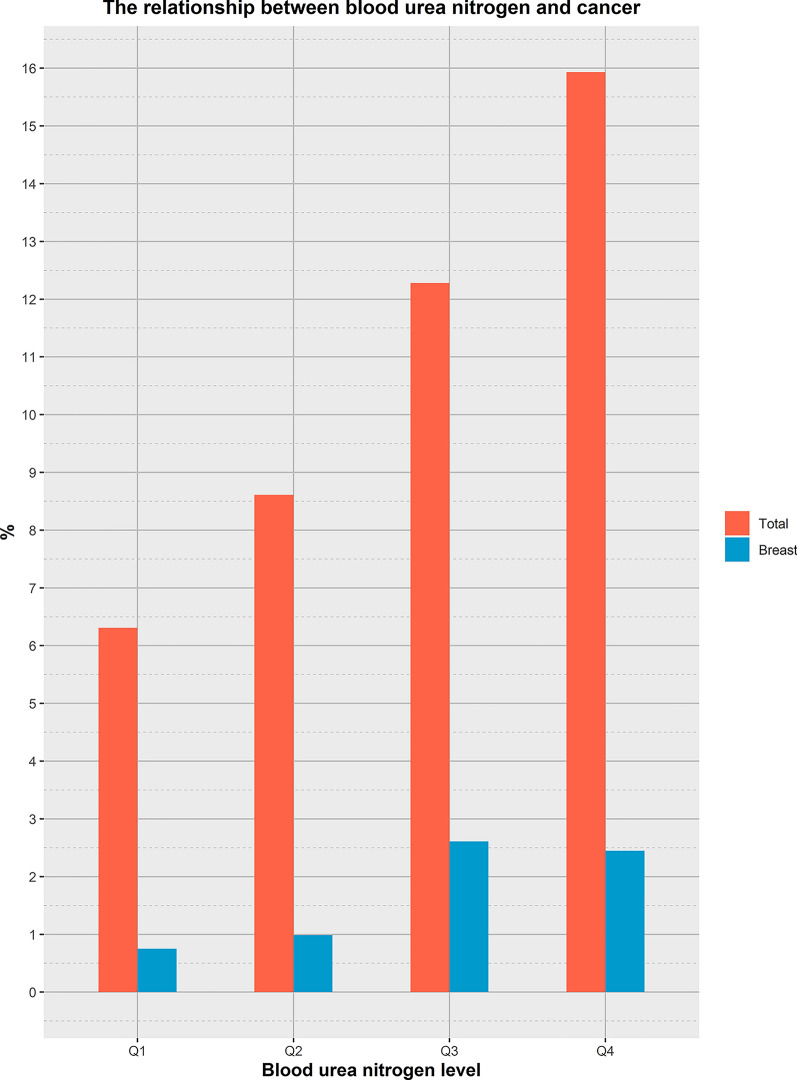
Distribution trend of breast cancer prevalence in different BUN level

To explore the relationship between BUN level and the prevalence of different cancers, we performed a regression analysis of the relationship between BUN level and cancer species after adjusting for age, race, PIR, smoking, alcohol consumption, waist circumference, energy intake, hypertension, diabetes, gender, education, BMI, and duration of moderate exercise (Fig. 2). The analysis showed a positive correlation between BUN level and prevalence in breast cancer patients (*P* < 0.01), and those with high BUN level were more likely to develop breast cancer (OR: 1.05, 95% CI 1.02–1.07). However, there was no association between BUN level and cancer prevalence in colon cancer (*P* = 0.77), melanoma (*P* = 0.07), prostate cancer (*P* = 0.67), uterine cancer (*P* = 0.20), and skin cancer (*P* = 0.43). To further verify the relationship between BUN level and breast cancer, we analyzed the relationship between the amount of BUN into four levels based on median and interquartile spacing. As shown in Fig. 3, the distribution trend of breast cancer prevalence among different BUN level was shown: the prevalence of breast cancer gradually increases with the increase of BUN level.

### Subgroup analysis

For all cancers, the results of subgroup analysis are shown in Table 3. Compared with the people with low level of BUN, the people with high level of BUN had a higher prevalence of cancer in patients aged < 65 years old, women (*P* < 0.01), senior high school education (*P* < 0.01), BMI ≥ 25 (*P* < 0.04), never smoking (*P* < 0.02), heavy drinking (*P* < 0.03) and non-diabetes (*P* < 0.01). In addition, the results of statistical analysis of breast cancer as a subgroup of all cancers (Table 4) showed that the prevalence of cancer was higher among age < 65 years (*P* < 0.01), non-Hispanic Whites (*P* = 0.01), non-Hispanic Blacks (*P* = 0.03), high school and higher education (*P* = 0.02, *P* = 0.03), PIR < 2.9 (*P* = 0.03), ever smokers (*P* < 0.01), moderate alcohol consumption (*P* = 0.02), small waist circumference (*P* = 0.01), low energy intake (*P* = 0.02), non-diabetic (*P* = 0.01), and non-hypertensive (*P* < 0.01) populations, BUN level was positively associated with breast cancer prevalence.

**Table 3:** Results of the analysis on subgroups of all cancers

Subgroup variable	OR (95% CI)	*P* value
Age ~ %		
< 65	1.05 (1.01, 1.08)	< 0.01
≥ 65	1.00 (0.98, 1.03)	0.75
Gender ~ %		
Male	1.01 (0.98, 1.03	0.52
Female	1.03 (1.01, 1.06)	0.01
Race ~ %		
Non-Hispanic White	1.02 (1.00, 1.04)	0.09
Non-Hispanic Black	1.02 (0.99, 1.05)	0.13
Mexican American	0.99 (0.91, 1.09)	0.87
Other Hispanic	1.01 (0.95, 1.07)	0.81
Other race	1.05 (0.96, 1.15)	0.28
Education level ~ %		
Less than high school	0.96 (0.91, 1.01)	0.12
High school	1.01 (0.97, 1.05)	0.55
More than high school	1.03 (1.01, 1.05)	0.01
Family PIR ~ %		
< 2.9	1.02 (0.99, 1.05)	0.22
≥ 2.9	1.02 (1.00, 1.05)	0.07
BMI ~ kg/m^2^		
< 25	1.01 (0.98, 1.04)	0.58
≥ 25	1.02 (1.00, 1.05)	0.04
Smoking behavior ~ %		
Never	1.03 (1.00, 1.06)	0.02
Former	1.02 (0.98, 1.05)	0.33
Now	0.98 (0.92, 1.05)	0.56
Alcohol consumption ~ %		
Never	1.04 (0.97, 1.10)	0.25
Former	1.01 (0.97, 1.05)	0.62
Mild	1.01 (0.99, 1.04)	0.37
Moderate	1.03 (0.98, 1.09)	0.24
Heavy	1.07 (1.01, 1.14)	0.03
Waist ~ cm		
< 97	1.03 (1.00, 1.06)	0.07
≥ 97	1.02 (0.99, 1.05)	0.19
Energy intake ~ kcal		
< 1950	1.03 (1.00, 1.05)	0.02
≥ 1950	1.01 (0.98, 1.05)	0.4
Hypertension ~ %		
Yes	1.03 (1.00, 1.05)	0.05
No	1.01 (0.98, 1.05)	0.48
Diabetes ~ %		
Yes	0.98 (0.95, 1.02)	0.38
No	1.04 (1.02, 1.06)	< 0.01

**Table 4:** Table of analysis results between breast cancer and BUN in cancer subgroup

Subgroup variable	Cancer = breast	
	OR (95% CI)	*P* value
Age ~ %		
< 65	1.10 (1.04, 1.15)	< 0.01
≥ 65	1.00 (0.95, 1.05)	0.99
Race ~ %		
Non-Hispanic White	1.05 (1.01, 1.09)	0.01
Non-Hispanic Black	1.05 (1.00, 1.10)	0.03
Mexican American	1.06 (0.82, 1.36)	0.66
Other Hispanic	0.99 (0.77, 1.28)	0.96
Other race	1.07 (0.92, 1.25)	0.35
Education level ~ %		
Less than high school	1.00 (0.92, 1.10)	0.93
High school	1.09 (1.02, 1.18)	0.02
More than high school	1.04 (1.00, 1.08)	0.03
Family PIR ~ %		
< 2.9	1.04 (1.00, 1.09)	0.03
≥ 2.9	1.04 (1.00, 1.08)	0.05
BMI ~ kg/m^2^		
< 25	1.08 (1.00, 1.17)	0.05
≥ 25	1.04 (1.00, 1.08)	0.06
Smoking behavior ~ %		
Never	1.04 (1.00, 1.07)	0.06
Former	1.09 (1.04, 1.14)	< 0.01
Now	1.07 (0.87, 1.31)	0.52
Alcohol consumption ~ %		
Never	1.05 (0.98, 1.14)	0.18
Former	0.97 (0.87, 1.07)	0.5
Mild	1.04 (0.99, 1.10)	0.11
Moderate	1.11 (1.02, 1.20)	0.02
Heavy	1.24 (0.98, 1.57)	0.08
Waist ~ cm		
< 97	1.07 (1.02, 1.13)	0.01
≥ 97	1.01 (0.97, 1.07)	0.58
Energy intake ~ kcal		
< 1950	1.04 (1.01, 1.08)	0.02
≥ 1950	1.06 (0.98, 1.14)	0.15
Hypertension ~ %		
Yes	1.03 (0.99, 1.07)	0.18
No	1.10 (1.02, 1.18)	0.01
Diabetes ~ %		
Yes	1.00 (0.93, 1.07)	0.93
No	1.06 (1.02, 1.10)	< 0.01

## Discussion

In order to study the relationship between BUN level and cancer incidence, we made a statistical analysis of the population data selected from the NHANES database. Through the adjustment and analysis of age, race, PIR, smoking, drinking, waistline, energy intake, hypertension, diabetes, sex, education, BMI, moderate exercise time and other variables, it was found that people with higher level of BUN had an increased risk of cancer, especially breast cancer. We speculated that the correlation between BUN level and cancer was caused by substance metabolism. BUN was the main end product of human protein metabolism, and the change of its concentration had a great influence on the metabolic balance in the body. Changes in protein metabolism in the body led to changes in the metabolism of other nutrients in the body, such as sugar metabolism, fat metabolism [23, 24]. Diet, hypermetabolic state of the organism, kidney disease, liver disease, and blood volume deficiency all had an impact on the metabolic level of BUN, and our study factors included these variables [25].

At present, most studies showed that the development of cancer was accompanied by specific changes in metabolism [26–30]. Metabolic disorders and cancer were both diseases with high prevalence, and the relationship between them had been widely studied. People with metabolic disorders were more likely to develop cancer [31]. In addition, metabolic abnormalities also altered the metabolic profile of cancer cells and the development of cancer through some signaling pathways or gene regulation [32]. When the protein metabolism in the body was disturbed, the BUN level would also change. At the same time, the metabolism level of other nutrients in the body would also play a change, thus the metabolic state of the whole body would change. In a study on the relationship between lipid metabolism and cancer, it was found that lipid metabolites could act as signal molecules of cancer cell activity, and could be used as nutrients to promote cancer cell proliferation, differentiation and migration [33, 34]. What is even more significant was that the relationship between lipid metabolism and breast cancer had been found to be very close according to numerous studies. The substances of lipid metabolism played an important role in the development, progression and metastasis of the breast [35–37]. This showed a certain correlation with our research results. Although the effect of BUN on lipid metabolism was not clear, the relationship between them must be interaction and interaction. We speculated that when the BUN level in the body changed, lipid metabolism as well as other substance metabolism changed, which led to a high risk of cancer development, especially the risk of breast cancer. Combined with our analysis results, BUN may have a greater effect on lipid metabolism, which leads to a high correlation between BUN level and breast cancer. As an important biomarker in metabolomics, BUN was of great significance to the metabolism of the body. More importantly, the close association between metabolomics and cancer had important implications for the diagnosis, treatment, and prognosis of cancer [38–40]. However, the effect and mechanism of BUN metabolism on the metabolism of other substances need to be further studied. The relationship between substance metabolism is an extremely complex process, which requires long-term research, but it is of great significance for human beings to overcome cancer.

## Conclusion

This study confirmed the correlation between BUN level and cancer prevalence, and that high BUN level was an important risk factor for cancer occurrence, especially breast cancer. The relationship between BUN level and cancer prevalence may be explained by changes in substance metabolism, however, this is a complex and long process. With the discovery of more risk factors related to the occurrence of cancer, the early detection and treatment of cancer will be better realized, and even the cure of cancer will be achieved finally.

## Data Availability

The data set supporting the conclusion of this article is included within the article.
